# Bromidobis(1,10-phenanthroline-κ^2^ *N*,*N*′)copper(II) dicyanamidate

**DOI:** 10.1107/S1600536810037979

**Published:** 2010-09-30

**Authors:** Ivan Potočňák, Zuzana Pravcová, Dmytro Rak

**Affiliations:** a Department of Inorganic Chemistry, Faculty of Science, P.J. Šafárik University, Moyzesova 11, SK-041 54 Košice, Slovakia

## Abstract

The title compound, [CuBr(C_12_H_8_N_2_)_2_][N(CN)_2_], is formed of discrete [CuBr(phen)_2_]^+^ complex cations and uncoordinated [N(CN)_2_]^−^ anions (phen is 1,10-phenanthroline). The Cu atom is five-coordinated in a distorted trigonal-bipyramidal geometry by two phen mol­ecules and one bromide ligand, which coordinates in the equatorial plane at a distance of 2.5228 (4) Å and lying along with the Cu and the amide N atoms on a twofold rotation axis. The two axial Cu—N distances [1.9926 (15) Å] are slightly shorter than the two equatorial Cu—N bonds [2.0979 (15) Å]. The structure is stabilized by a weak C—H⋯N hydrogen bond, with a cyanide N atom of the dicyanamide ligand as an acceptor, and π–π inter­actions between nearly parallel phenyl and pyridine rings of two adjacent phen mol­ecules [centroid–centroid distance = 3.589 (1) Å], and between π electrons of the dicyanamide anion and the pyridine ring [N⋯*Cg*(pyridine) = 3.511 (3) Å; C—N⋯*Cg*(pyridine) = 80.2 (2)°].

## Related literature

For structures containing [Cu(phen)_2_Br]^+^ cations, see: Murphy *et al.* (1998 ▶); Parker *et al.* (1994 ▶); Lu *et al.* (2004 ▶). For penta­coordinated Cu(II) in [Cu(*L*)_2_dca]*Y* complexes [*L* = 1,10- phenanthroline (phen) and 2,2′-bipyridine (bpy), *Y* is a monovalent anion], see: Potočňák *et al.* (2005 ▶, 2006 ▶, 2008*a* ▶,*b* ▶). For π–π inter­actions, see: Janiak (2000 ▶). For the τ parameter, see: Addison *et al.* (1984 ▶). For a description of the Cambridge Structural Database, see: Allen (2002 ▶). For reference bond lengths, see: Jolly (1991 ▶).
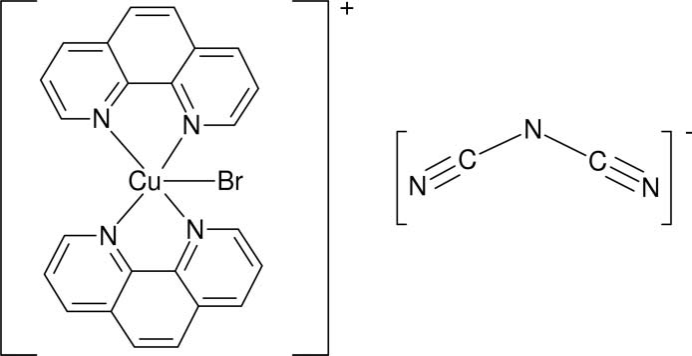


## Experimental

### 

#### Crystal data

[CuBr(C_12_H_8_N_2_)_2_]C_2_N_3_
*M* *_r_* = 569.91
Monoclinic, 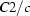

*a* = 15.2317 (4) Å
*b* = 10.8270 (3) Å
*c* = 14.7408 (5) Å
β = 114.030 (4)°
*V* = 2220.27 (11) Å^3^
*Z* = 4
Mo *K*α radiation
μ = 2.82 mm^−1^
*T* = 293 K
0.68 × 0.17 × 0.09 mm

#### Data collection

Oxford Diffraction Xcalibur2 CCD diffractometer
Absorption correction: analytical (*CrysAlis RED*; Oxford Diffraction, 2007 ▶) *T*_min_ = 0.328, *T*_max_ = 0.819
11517 measured reflections
2182 independent reflections
1799 reflections with *I* > 2σ(*I*)
*R*_int_ = 0.026

#### Refinement

*R*[*F* ^2^ > 2σ(*F* ^2^)] = 0.021
*wR*(*F* ^2^) = 0.062
*S* = 1.06
2182 reflections
160 parameters
H-atom parameters constrained
Δρ_max_ = 0.29 e Å^−3^
Δρ_min_ = −0.43 e Å^−3^

### 

Data collection: *CrysAlis CCD* (Oxford Diffraction, 2007 ▶); cell refinement: *CrysAlis RED* (Oxford Diffraction, 2007 ▶); data reduction: *CrysAlis RED*; program(s) used to solve structure: *SHELXS97* (Sheldrick, 2008 ▶); program(s) used to refine structure: *SHELXL97* (Sheldrick, 2008 ▶); molecular graphics: *DIAMOND* (Brandenburg, 2001 ▶); software used to prepare material for publication: *SHELXL97*.

## Supplementary Material

Crystal structure: contains datablocks I, global. DOI: 10.1107/S1600536810037979/kp2274sup1.cif

Structure factors: contains datablocks I. DOI: 10.1107/S1600536810037979/kp2274Isup2.hkl

Additional supplementary materials:  crystallographic information; 3D view; checkCIF report

## Figures and Tables

**Table 1:** Hydrogen-bond geometry (Å, °)

*D*—H⋯*A*	*D*—H	H⋯*A*	*D*⋯*A*	*D*—H⋯*A*
C22—H22⋯N2^i^	0.93	2.60	3.346 (3)	137

Symmetry code: (i) 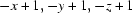.

**Fig. 1.**
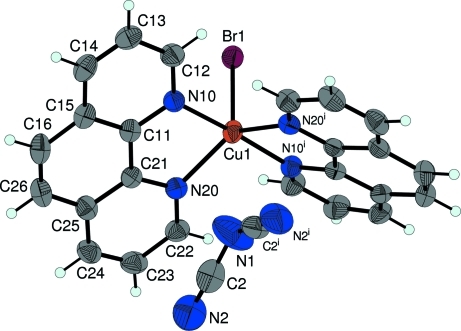
The molecular structure of the title compound, with 50% probability displacement ellipsoids for non-H atoms. [Symmetry code: (i) 1 - x, y, 1/2 - z]

**Fig. 2.**
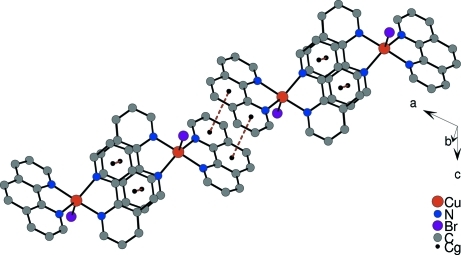
π-π interactions (dashed lines) between nearly parallel phenyl and pyridine rings in (I).

**Fig. 3.**
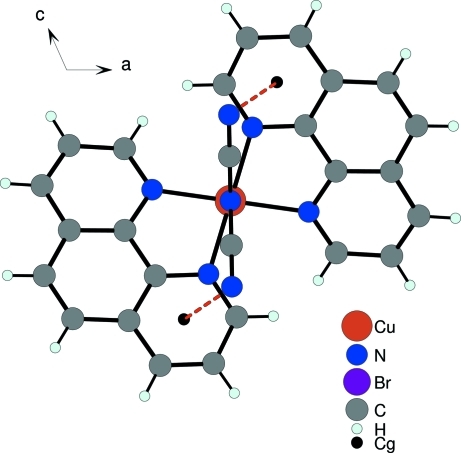
π-π Interactions (dashed lines) between dca and pyridine ring in (I).

## supplementary crystallographic information

### Comment

The molecular structures of five-coordinated [Cu(*L*)_2_*X*]Y complexes (*L* is 1,10-phenanthroline (phen) or 2,2'-bipyridine (bpy), *X* and Y are monovalent anions) exhibit an extensive variability ranging from trigonal bipyramid to square pyramidal stereochemistries, with majority complexes displaying a structure which is intermediate between these two extremes (Allen, 2002). In our previous work we have used dicyanamide (dca) within our study on the spectral-structural correlations of penta-coordinated [Cu(*L*)_2_dca]Y complexes (*L* = 1,10-phenanthroline (phen) and 2,2'-bipyridine (bpy) and Y is a monovalent anion) (Potočňák *et al.*, 2008*a*; Potočňák *et al.*, 2005). Within this study we also tried to prepare a [Cu(phen)_2_dca]Cl complex but the synthesis resulted in the complex with exchanged anions, [Cu(phen)_2_Cl]dca (Potočňák *et al.*, 2006). With the aim to continue in this work and with the hope that a larger anion, namely Br^-^, enables dca to enter the inner coordination sphere of the copper atom we decided to prepare a [Cu(phen)_2_dca]Br complex. Nevertheless, X-ray structure analysis revealed that the prepared complex is [Cu(phen)_2_Br]dca (I) and here we present its structure (Fig. 1). The crystal and molecular symmetry has a twofold axis parallel to the *b* axis through the copper, bromine and amide nitrogen atoms. The same symmetry was observed in the [Cu(phen)_2_Cl]dca (II) (Potočňák *et al.*, 2006) and [Cu(phen)_2_Br]ClO_4_ (III) complexes (Parker *et al.*, 1994) (the twofold axis passes through chlorine atoms of the chloride and perchlorate anions, respectively) which are isostructural to (I). Structures of other [Cu(phen)_2_Br]Y complexes are described by Murphy *et al.* (1998). The structure of (I) is formed by [Cu(phen)_2_Br] cations and dca anions. The structure of the cation consists of two phen molecules and one bromide ion coordinated to a copper(II) atom in a five-coordinate distorted trigonal bipyramidal geometry as evidenced by the τ parameter of of Addison *et al.* (1984); the value being 70.9 (69.6 and 94.0 for (II) and (III), respectively) (the τ parameter is 100 for an ideal trigonal bipyramid and 0 for an ideal square pyramid). Each of the two phen molecules possesses one nitrogen atom (N20) occupying an equatorial position and one nitrogen atom (N10) coordinated in an axial position. The two axial Cu1—N10 bonds are almost collinear (Table 1) and are shorter by 0.105 Å than the two equatorial Cu1—N20 bonds, which is a feature generally observed for compounds with [Cu(phen)_2_*X*]^+^ cations (Murphy *et al.*, 1998, Lu *et al.*, 2004, Parker *et al.*, 1994, Potočňák *et al.*, 2005, Potočňák *et al.*, 2008**a*,b*). Aromatic rings of phen molecules are nearly planar; the largest deviation of atoms from their mean planes is 0.112 (2) Å and the bond distances and angles are normal. The bromide ion occupies the third equatorial position at a distance of 2.5228 (4) Å, which is slightly longer than corresponding distances observed in other [Cu(phen)_2_Br]Y complexes (Murphy *et al.*, 1998, Parker *et al.*, 1994).

Each distinct [Cu(phen)_2_Br] cation has a separate dca anion, which is settled under the umbrella of the copper and the two phenanthrolines. The N_cyanide_≡C (1.145 (3) Å) as well as the N_amide_=C distance (1.288 (4) Å) are usual for triple N≡C (1.15 Å) and double N=C (1.27 Å) bonds (Jolly, 1991). The bond angle around cyanido C2 atom is, as expected, nearly linear (174.8 (3)°) and the angle around amide N1 atom is consistent with *sp*^2^ hybridization (120.7 (4)°). All mentioned values of bonds and angles are close to the values observed in the previously mentioned [Cu(*L*)_2_dca]Y compounds.

The structure of (I) is stabilised by a weak C—H···N hydrogen bond with cyanide N2 atom as acceptor (Table 3). The next stabilization comes from two kinds of π-π interactions (Janiak, 2000). There are face to face π-π interactions between nearly parallel phenyl and pyridine rings of two adjacent phen molecules (Fig. 2) as evidenced by the distance of *Cg*(phenyl)-*Cg*(pyridine)^i^ = 3.589 (1) Å and by the angle between phenyl ring normal and vector connecting the two centroids of 9.48° (i = 1.5 - *x*, 1.5 - *y*, 1 - *z*). The next type of π-π interaction is an interaction between π electrons of the dca anion and the pyridine ring. This interaction is described by the C2—N2···*Cg*(pyridine) angle of 80.2 (2)° and by the N2···*Cg*(pyridine) distance of 3.511 (3) Å (Fig. 3).

### Experimental

The title compound was prepared by chance during our attempts to prepare [Cu(phen)_2_(dca)]Br compound. Crystals of (I) were prepared by mixing a 0.1 *M* aqueous solution of CuBr_2_ (5 ml; 0.5 mmol) with a 0.1 *M* ethanolic solution of phen (10 ml; 1 mmol). To the resulting dark green solution, a 0.1 *M* ethanolic solution of NaN(CN)_2_ (5 ml; 0.5 mmol) was added (all solutions were warmed before mixing). After a few days, green crystals were filtered off and dried in air.

### Refinement

Anisotropic displacement parameters were refined for all non-H atoms. H-atoms were positioned geometrically and refined as riding atoms, with C—H = 0.93 Å and *U*_iso_(H) = 1.2*U*_eq_(C).

### Crystal data

**Table:**

[CuBr(C_12_H_8_N_2_)_2_]C_2_N_3_	*F*(000) = 1140
*M**_r_* = 569.91	*D*_x_ = 1.705 Mg m^−^^3^
Monoclinic, *C*2/*c*	Mo *K*α radiation, λ = 0.71069 Å
Hall symbol: -C 2yc	Cell parameters from 7148 reflections
*a* = 15.2317 (4) Å	θ = 3.0–29.5°
*b* = 10.8270 (3) Å	µ = 2.82 mm^−^^1^
*c* = 14.7408 (5) Å	*T* = 293 K
β = 114.030 (4)°	Prism, green
*V* = 2220.27 (11) Å^3^	0.68 × 0.17 × 0.09 mm
*Z* = 4	

### Data collection

**Table:**

Oxford Diffraction Xcalibur2 CCD diffractometer	2182 independent reflections
Radiation source: Enhance (Mo) X-ray Source	1799 reflections with *I* > 2σ(*I*)
graphite	*R*_int_ = 0.026
Detector resolution: 8.3438 pixels mm^-1^	θ_max_ = 26.0°, θ_min_ = 3.0°
Rotation method data acquisition using ω scans	*h* = −18→18
Absorption correction: analytical (*CrysAlis RED*; Oxford Diffraction, 2007)	*k* = −13→13
*T*_min_ = 0.328, *T*_max_ = 0.819	*l* = −18→18
11517 measured reflections	

### Refinement

**Table:**

Refinement on *F*^2^	Primary atom site location: structure-invariant direct methods
Least-squares matrix: full	Secondary atom site location: difference Fourier map
*R*[*F*^2^ > 2σ(*F*^2^)] = 0.021	Hydrogen site location: inferred from neighbouring sites
*wR*(*F*^2^) = 0.062	H-atom parameters constrained
*S* = 1.06	*w* = 1/[σ^2^(*F*_o_^2^) + (0.0401*P*)^2^] where *P* = (*F*_o_^2^ + 2*F*_c_^2^)/3
2182 reflections	(Δ/σ)_max_ < 0.001
160 parameters	Δρ_max_ = 0.29 e Å^−^^3^
0 restraints	Δρ_min_ = −0.43 e Å^−^^3^

### Special details

**Table:**

Experimental. CrysAlisPro, Oxford Diffraction Ltd., Analytical numeric absorption correction using a multifaceted crystal model based on expressions derived by R.C. Clark & J.S. Reid. (Clark, R. C. & Reid, J. S. (1995). Acta Cryst. A51, 887-897
Geometry. All e.s.d.'s (except the e.s.d. in the dihedral angle between two l.s. planes) are estimated using the full covariance matrix. The cell e.s.d.'s are taken into account individually in the estimation of e.s.d.'s in distances, angles and torsion angles; correlations between e.s.d.'s in cell parameters are only used when they are defined by crystal symmetry. An approximate (isotropic) treatment of cell e.s.d.'s is used for estimating e.s.d.'s involving l.s. planes.
Refinement. Refinement of *F*^2^ against ALL reflections. The weighted *R*-factor *wR* and goodness of fit *S* are based on *F*^2^, conventional *R*-factors *R* are based on *F*, with *F* set to zero for negative *F*^2^. The threshold expression of *F*^2^ > σ(*F*^2^) is used only for calculating *R*-factors(gt) *etc*. and is not relevant to the choice of reflections for refinement. *R*-factors based on *F*^2^ are statistically about twice as large as those based on *F*, and *R*- factors based on ALL data will be even larger.

### Fractional atomic coordinates and isotropic or equivalent isotropic displacement parameters (Å^2^)

**Table:**

	*x*	*y*	*z*	*U*_iso_*/*U*_eq_	
Cu1	0.5000	0.72646 (3)	0.2500	0.03323 (11)	
N10	0.62123 (11)	0.71984 (14)	0.22918 (11)	0.0327 (4)	
N20	0.59053 (11)	0.64969 (13)	0.38697 (12)	0.0321 (4)	
Br1	0.5000	0.95947 (2)	0.2500	0.03604 (10)	
C11	0.69628 (13)	0.66725 (15)	0.30537 (13)	0.0308 (4)	
C12	0.63710 (15)	0.76473 (19)	0.15301 (15)	0.0396 (5)	
H12	0.5869	0.8039	0.1017	0.047*	
C13	0.72584 (16)	0.75547 (19)	0.14697 (16)	0.0434 (5)	
H13	0.7342	0.7877	0.0925	0.052*	
C14	0.80010 (15)	0.6989 (2)	0.22157 (16)	0.0415 (5)	
H14	0.8590	0.6898	0.2173	0.050*	
C15	0.78767 (14)	0.65401 (16)	0.30513 (15)	0.0348 (4)	
C16	0.86264 (14)	0.6003 (2)	0.38968 (15)	0.0445 (5)	
H16	0.9231	0.5888	0.3894	0.053*	
C21	0.67959 (13)	0.63020 (16)	0.39026 (14)	0.0295 (4)	
C22	0.57510 (15)	0.62008 (19)	0.46690 (15)	0.0380 (5)	
H22	0.5139	0.6310	0.4651	0.046*	
C23	0.64771 (16)	0.57311 (19)	0.55360 (15)	0.0443 (5)	
H23	0.6347	0.5552	0.6086	0.053*	
C24	0.73686 (16)	0.55385 (18)	0.55692 (15)	0.0428 (5)	
H24	0.7853	0.5224	0.6142	0.051*	
C25	0.75581 (14)	0.58155 (17)	0.47343 (14)	0.0356 (4)	
C26	0.84725 (16)	0.56610 (19)	0.46978 (16)	0.0455 (5)	
H26	0.8974	0.5316	0.5240	0.055*	
N1	0.5000	0.4095 (4)	0.2500	0.0987 (14)	
C2	0.5311 (2)	0.3506 (2)	0.3332 (2)	0.0560 (6)	
N2	0.56044 (18)	0.3068 (3)	0.41052 (18)	0.0749 (7)	

### Atomic displacement parameters (Å^2^)

**Table:**

	*U*^11^	*U*^22^	*U*^33^	*U*^12^	*U*^13^	*U*^23^
Cu1	0.02710 (19)	0.0454 (2)	0.03011 (19)	0.000	0.01467 (14)	0.000
N10	0.0319 (9)	0.0372 (9)	0.0308 (9)	0.0010 (7)	0.0146 (7)	0.0012 (7)
N20	0.0348 (9)	0.0320 (8)	0.0295 (9)	−0.0022 (7)	0.0131 (7)	−0.0029 (6)
Br1	0.03279 (16)	0.03849 (17)	0.03668 (17)	0.000	0.01399 (12)	0.000
C11	0.0322 (10)	0.0290 (10)	0.0307 (10)	−0.0017 (8)	0.0122 (8)	−0.0071 (8)
C12	0.0405 (12)	0.0453 (12)	0.0371 (11)	0.0029 (9)	0.0200 (9)	0.0040 (9)
C13	0.0479 (13)	0.0485 (12)	0.0450 (12)	−0.0028 (10)	0.0303 (11)	−0.0002 (10)
C14	0.0358 (11)	0.0456 (11)	0.0522 (13)	−0.0021 (10)	0.0271 (10)	−0.0103 (10)
C15	0.0323 (10)	0.0347 (10)	0.0397 (11)	−0.0015 (8)	0.0169 (8)	−0.0103 (8)
C16	0.0288 (11)	0.0509 (12)	0.0500 (14)	0.0032 (10)	0.0121 (10)	−0.0099 (11)
C21	0.0320 (10)	0.0265 (9)	0.0290 (10)	−0.0029 (7)	0.0114 (8)	−0.0056 (7)
C22	0.0418 (12)	0.0423 (11)	0.0338 (11)	−0.0044 (9)	0.0194 (9)	−0.0004 (9)
C23	0.0558 (14)	0.0457 (13)	0.0304 (11)	−0.0075 (10)	0.0164 (10)	0.0021 (9)
C24	0.0463 (13)	0.0424 (12)	0.0299 (11)	−0.0039 (9)	0.0055 (10)	0.0018 (9)
C25	0.0368 (11)	0.0319 (10)	0.0318 (10)	−0.0020 (9)	0.0075 (9)	−0.0059 (9)
C26	0.0346 (11)	0.0499 (13)	0.0413 (12)	0.0059 (9)	0.0048 (10)	−0.0021 (10)
N1	0.139 (4)	0.065 (2)	0.055 (2)	0.000	0.001 (2)	0.000
C2	0.0556 (15)	0.0599 (15)	0.0556 (16)	−0.0062 (12)	0.0257 (13)	−0.0226 (14)
N2	0.0795 (17)	0.1034 (18)	0.0465 (14)	−0.0076 (15)	0.0304 (12)	−0.0144 (13)

### Geometric parameters (Å, °)

**Table:**

Cu1—N10^i^	1.9926 (15)	C14—H14	0.9300
Cu1—N10	1.9926 (15)	C15—C16	1.427 (3)
Cu1—N20^i^	2.0979 (15)	C16—C26	1.346 (3)
Cu1—N20	2.0979 (15)	C16—H16	0.9300
Cu1—Br1	2.5228 (4)	C21—C25	1.403 (3)
N10—C12	1.333 (3)	C22—C23	1.401 (3)
N10—C11	1.359 (2)	C22—H22	0.9300
N20—C22	1.331 (2)	C23—C24	1.355 (3)
N20—C21	1.354 (2)	C23—H23	0.9300
C11—C15	1.401 (3)	C24—C25	1.406 (3)
C11—C21	1.432 (3)	C24—H24	0.9300
C12—C13	1.394 (3)	C25—C26	1.425 (3)
C12—H12	0.9300	C26—H26	0.9300
C13—C14	1.361 (3)	N1—C2	1.288 (4)
C13—H13	0.9300	N1—C2^i^	1.288 (4)
C14—C15	1.407 (3)	C2—N2	1.145 (3)
			
N10^i^—Cu1—N10	175.88 (9)	C15—C14—H14	120.0
N10^i^—Cu1—N20^i^	81.19 (6)	C11—C15—C14	117.07 (18)
N10—Cu1—N20^i^	97.16 (6)	C11—C15—C16	118.83 (19)
N10^i^—Cu1—N20	97.16 (6)	C14—C15—C16	124.07 (19)
N10—Cu1—N20	81.19 (6)	C26—C16—C15	121.0 (2)
N20^i^—Cu1—N20	133.32 (8)	C26—C16—H16	119.5
N10^i^—Cu1—Br1	92.06 (4)	C15—C16—H16	119.5
N10—Cu1—Br1	92.06 (4)	N20—C21—C25	123.38 (18)
N20^i^—Cu1—Br1	113.34 (4)	N20—C21—C11	117.18 (16)
N20—Cu1—Br1	113.34 (4)	C25—C21—C11	119.38 (18)
C12—N10—C11	117.96 (17)	N20—C22—C23	122.4 (2)
C12—N10—Cu1	127.88 (14)	N20—C22—H22	118.8
C11—N10—Cu1	114.09 (12)	C23—C22—H22	118.8
C22—N20—C21	117.78 (17)	C24—C23—C22	119.7 (2)
C22—N20—Cu1	131.57 (14)	C24—C23—H23	120.1
C21—N20—Cu1	110.64 (12)	C22—C23—H23	120.1
N10—C11—C15	122.90 (18)	C23—C24—C25	119.70 (19)
N10—C11—C21	116.79 (16)	C23—C24—H24	120.1
C15—C11—C21	120.25 (17)	C25—C24—H24	120.1
N10—C12—C13	122.74 (19)	C21—C25—C24	116.94 (19)
N10—C12—H12	118.6	C21—C25—C26	118.98 (19)
C13—C12—H12	118.6	C24—C25—C26	124.06 (19)
C14—C13—C12	119.3 (2)	C16—C26—C25	121.52 (19)
C14—C13—H13	120.3	C16—C26—H26	119.2
C12—C13—H13	120.3	C25—C26—H26	119.2
C13—C14—C15	119.94 (19)	C2—N1—C2^i^	120.7 (4)
C13—C14—H14	120.0	N2—C2—N1	174.8 (3)

Symmetry codes: (i) −*x*+1, *y*, −*z*+1/2.

### Hydrogen-bond geometry (Å, °)

**Table:**

*D*—H···*A*	*D*—H	H···*A*	*D*···*A*	*D*—H···*A*
C22—H22···N2^ii^	0.93	2.60	3.346 (3)	137

Symmetry codes: (ii) −*x*+1, −*y*+1, −*z*+1.
